# Combined Anti-Inflammatory and Anti-AGE Drug Treatments Have a Protective Effect on Intervertebral Discs in Mice with Diabetes

**DOI:** 10.1371/journal.pone.0064302

**Published:** 2013-05-17

**Authors:** Svenja Illien-Junger, Fabrizio Grosjean, Damien M. Laudier, Helen Vlassara, Gary E. Striker, James C. Iatridis

**Affiliations:** 1 Leni & Peter May Dept. of Orthopaedics, Mount Sinai School of Medicine, New York, New York, United States of America; 2 Department of Geriatrics and Palliative Care, Division of Experimental Diabetes and Aging Mount Sinai School of Medicine, New York, New York, United States of America; 3 Unit of Dialysis, Nephrology and Transplantation, Foundation Policlinico San Matteo IRCCS, Square Golgi, Pavia, Italy; 4 Department of Geriatrics and Palliative Care, Division of Experimental Diabetes and Aging, and Division of Nephrology, Department of Medicine, Mount Sinai School of Medicine, New York, New York, United States of America; Consiglio Nazionale delle Ricerche, Italy

## Abstract

**Objective:**

Diabetes and low back pain are debilitating diseases and modern epidemics. Diabetes and obesity are also highly correlated with intervertebral disc (IVD) degeneration and back pain. Advanced-glycation-end-products (AGEs) increase reactive-oxygen-species (ROS) and inflammation, and are one cause for early development of diabetes mellitus. We hypothesize that diabetes results in accumulation of AGEs in spines and associated spinal pathology via increased catabolism. We present a mouse model showing that: 1) diabetes induces pathological changes to structure and composition of IVDs and vertebrae; 2) diabetes is associated with accumulation of AGEs, TNFα, and increased catabolism spinal structures; and 3) oral-treatments with a combination of anti-inflammatory and anti-AGE drugs mitigate these diabetes-induced degenerative changes to the spine.

**Methods:**

Three age-matched groups of ROP-Os mice were compared: non-diabetic, diabetic (streptozotocin (STZ)-induced), or diabetic mice treated with pentosan-polysulfate (anti-inflammatory) and pyridoxamine (AGE-inhibitor). Mice were euthanized and vertebra-IVD segments were analyzed by μCT, histology and Immunohistochemistry.

**Results:**

Diabetic mice exhibited several pathological changes including loss in IVD height, decreased vertebral bone mass, decreased glycosaminoglycan content and morphologically altered IVDs with focal deposition of tissues highly expressing TNFα, MMP-13 and ADAMTS-5. Accumulation of larger amounts of methylglyoxal suggested that AGE accumulation was associated with these diabetic degenerative changes. However, treatment prevented or reduced these pathological effects on vertebrae and IVD.

**Conclusion:**

This is the first study to demonstrate specific degenerative changes to nucleus pulposus (NP) morphology and their association with AGE accumulation in a diabetic mouse model. Furthermore, this is the first study to demonstrate that oral-treatments can inhibit AGE-induced ROS and inflammation in spinal structures and provide a potential treatment to slow progression of degenerative spine changes in diabetes. Since diabetes, IVD degeneration, and accumulation of AGEs are frequent consequences of aging, early treatments to reduce AGE-induced ROS and Inflammation may have broad public-health implications.

## Introduction

Low back pain is among the most common conditions requiring medical care with 40.5M patient visits in 2005, enormous annual medical costs ($193.9B and rising) and substantial lost productivity [1], [2]. Low back pain is often related to intervertebral disc (IVD) pathologies including degeneration [3], [4]. The causes for IVD degeneration are multifactorial and risk factors include heritability, rapid increase in weight, obesity and physical activity levels [4], [5], [6]. Importantly, juvenile disc degeneration was strongly associated with obesity, low back pain, increased low back pain intensity, and diminished physical and social functioning with a significant association of elevated body mass index with severity of disc degeneration [6], [7].

Diabetes Mellitus (DM) is a systemic disease reaching epidemic proportions on world-wide basis, and DM causes degenerative changes of most of the body organ systems [8] including the nervous, cardiovascular, kidney, and the musculoskeletal systems [9], [10], [11], [12], [13]. The WHO reports that more than 346 million people worldwide have diabetes (http://www.who.int/mediacentre/factsheets/fs312/en/). In the United States, 26.5 million are affected by diabetes mellitus type 2 (>95% of all diagnosed diabetic cases) 67 million are estimated to have pre-diabetes, numbers that have tripled since the 1980s (http://www.cdc.gov/obesity/childhood/index.html). Alarmingly, at least 154,000 children and adolescents in the U.S. have DM (based on 2002–2003 data), and the annual increase of people under 20 years having DM is approximately 24.3 of 100,000 per year with 15,000 diagnosed with type 1 DM and 3,700 diagnosed with type 2 DM [14], [15] (http://www.cdc.gov/obesity/childhood/index.html). Thus, diabetes affects people who are actively growing and those who have reached skeletal maturity.

Despite the tremendous medical and societal impact of DM and IVD degeneration, there is remarkably no direct evidence for a relationship between DM and spinal pathologies. DM is a risk factor for spinal stenosis, IVD degeneration, herniation, bone loss and accelerated aging [11], [16], [17], yet little is known about the inter-related pathophysiology and mechanisms for action that might link diabetes with spinal pathology. A prospective study revealed that patients which were operated on for lumbar IVD herniation had a higher prevalence of DM compared to patients operated on for other reasons, suggesting that DM is a predisposing factor for symptomatic disc disease [11], yet this relationship has not been well established. DM was observed to significantly affect the musculoskeletal tissue in diabetic and aging sand rats with changes in vertebrae and discs that were similar to human disc degeneration [13], [18].

Painful IVDs are known to be in a chronic pro-inflammatory state, and symptomatic human IVDs contain up-regulated pro-inflammatory cytokines [19], [20], [21]. Accumulation of advanced glycation end products (AGEs) with aging are also associated with increased levels of inflammation [22] and may be a further cause of the chronic pro-inflammatory state found in degenerated IVDs.

AGEs are formed by non-enzymatic glycation, resulting in oxidation of proteins and lipids. Diabetes, cardiovascular disease and chronic kidney disease are associated with increased levels of AGEs [23]. The major source of AGEs in both diabetics and normal individuals is the diet [24]. Nε-carboxymethyl lysine (CML) is formed by reactions of carbonyl precursors like methylglyoxal (MG) with carbohydrates and lipids, and the associated protein alterations are irreversible [25]. AGEs accumulate in the major long—lived proteins of the IVD including aggrecan and collagen preventing their repair and turnover [26]. Non-enzymatic crosslinking of human annulus fibrosus (AF) tissue with MG increased AF tensile stiffness [27], supporting the concept that accumulation of AGEs with aging and degeneration in the IVD increases stiffness and brittleness making the tissue more susceptible to mechanical damage [28]. Systemic levels of AGEs can be reduced by dietary restriction of AGE intake [29] or a non-absorbable drug that binds AGEs in the intestine and eliminates them in the stool, or through drugs that prevent AGE formation like aspirin [30], [31], benfotiamine [32], and pyridoxamine [33].

Two significant questions remain unanswered with respect to DM and IVD degeneration. First, does DM directly induce pathological changes to the IVD and vertebrae of the lumbar spine via AGE accumulation and increased inflammation? Second, can inhibition of the uptake or actions of AGEs by oral drugs slow the progression of degenerative changes to the spine? To our knowledge, this is the first study to investigate if DM has detrimental effects on vertebral and IVD morphology in a mouse model and if these changes can be blocked or reduced with oral medication. We hypothesized that a combination of anti-inflammatory and AGE inhibition drugs could mitigate DM induced vertebral and IVD pathologic changes by reducing the accumulation of AGEs. The results of this study demonstrated that diabetes induced vertebral bone loss and created structural defects and proteoglycan loss in the IVD. The STZ-induced Type 1 diabetes in mice resulted in accumulation of AGEs with an associated increase in the TNF-α and the production of catabolic proteins in the spine. The administration of oral anti-inflammatory and AGE inhibitors treatments (that are FDA approved for other uses) was shown to reduce accumulation of MG and catabolic proteins in the spine and to mitigate degenerative changes to the vertebrae and IVDs of diabetic mice.

## Materials and Methods

### Study design

**Figure 1:**
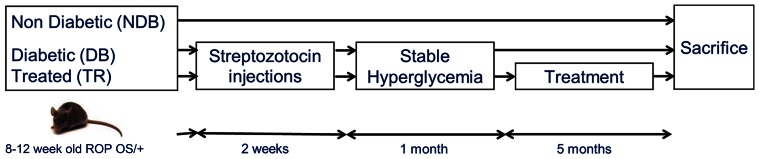
Study design.

Eight to twelve week old female ROP Os/+mice were maintained for 6.5 months. Mice were randomized in experimental & control mice (21 mice, n = 6–8 per group). Body weight was determined weekly; and in experimental mice (Db), diabetes was induced over a 2 week period using low dose streptozotocin injections (50 µg/g body wt of STZ). STZ injections induced hyperglycemia, which was controlled to <250 mg/dl with lente-insulin as needed. Non-diabetic control mice (NDb) obtained vehicle solution (citrate buffer) correspondingly. Glycemic levels were monitored, and mice that maintained blood glucose levels (>200 mg/dl) for 1 month were then randomized to two groups: Diabetic (Db) mice which were maintained without insulin treatment for another 5 months, and treated mice that underwent the same diabetes induction protocol but were given 5 months of treatments including: Pyridoxamine (PYR, an AGE inhibitor), and Pentosan Polysulphate (PPS, a broad acting anti-inflammatory). All diabetic mice received enalapril (EN, angiotensin converting enzyme inhibitor; Figure 1). This study was carried out in strict accordance with the recommendations in the Guide for the Care and Use of Laboratory Animals of the National Institutes of Health. (Department of Health, Education, and Welfare, NIH 78–23, 1996). All animal protocols were approved by the Mount Sinai Institutional Animal Care and Use Committee (protocol # 08-0108). All euthanasia was performed using carbon dioxide inhalation and all efforts were made to minimize suffering.

### Spine harvest and μCt

Following sacrifice, lumbar spines were dissected and fixed in z-fix. Before μCt, spines were washed in PBS. Trabecular bone of vertebrae and end plate as well as cortical bone analyses were performed using a Pre-Clinical Specimen Micro-computed Tomography system eXplore Locus SP. (GE Healthcare, London, Ontario, Canada). Lumbar vertebrae (L5) were used to measure bone structure as described below. Three-dimensional images of the entire lumbar spine were obtained with a short scan at an 8.7 micron voxel size (acquisition parameters: 400 views at 0.5 degree increment (9 pictures/view) 80 kVp, 80 uA, 3 second exposure). Cross-sections were analyzed for the amount of tissue (trabecular (Tb.) bone: Tissue Mineral Density, TMD) Tb. number (Tb.N.) Tb. Spaces (Tb.Sp.) and Bone volume fraction (BVF); cortical bone: marrow, cortical and total area Bone mineral density (BMD) and bone mineral content (BMC); total bone volume). Intervertebral disc and lumbar vertebrae (L4–L5) height measurements were calculated by specifying contour coordinates in Microview ABA 2.2 (GE healthcare) and calculating the difference between the coordinates via MATLAB 2010 (Mathworks). Contours were selected by manually approximating the contour outline with the polygon advanced ROI tool, then the “Shrink Wrap” tool was used to generate a finer outline of the contour with a resolution of 10 nodes/10 pixels. Vertebrae heights were automatically calculated by subtracting the y coordinates of nodes which are aligned on the x coordinate. Disc height index (DHI) and disc wedge index (DWI) were calculated based on sagittal IVD and vertebrae height measurements. The DHI reflects the disc height relative to the vertebrae (DHI = 2×IVD height/(L4+L5)) [34]; while the DWI correlates to the IVD shape. (DWI = disc height anterior/disc height posterior) [35]. A DWI value above 1 indicates that the anterior side of the disc is thicker than the posterior side, corresponding to a relative loss of the posterior disc height (i.e., hyperlordosis).

### Histomorphometric grading scheme

**S**ections of plastic embedded IVDs were used to score the degree of degeneration based on histological appearances of the NP. Safranin-O/fast green sections were graded within 8 parameters for signs of degeneration, based on a scoring system modified from Sive *et al* 2002 [36]. Scores within 3 categories were added together for a final score out of 8. The final scores were classified in 3 groups: no to minimal degeneration (score 0–2), moderate degeneration (score 3–5), severe degeneration (score 6–8). Three specimens per group were analyzed for the scoring system.

### Histology and immunohistochemistry

IVD-vertebrae **s**egments of L3-4 and L4-5 were decalcified and embedded either in plastic (n = 2–3 per group) or paraffin (n = 3–4 per group); Sections of plastic and Paraffin sections were used for Histology (Safranin-O/fast green). Immunohistochemistry for CML, MG, TNFα, ADAMTS-5 and MMP13 was performed on plastic embedded IVD-vertebrae sections.

### GAG content quantification

GAG intensity was measured on histology slides of plastic and paraffin embedded IVD slides. Regions of interest were defined manually (ImageJ; http://rsbweb.nih.gov/ij/) to include centrally located areas within the morphologically distinct AF and endplate regions. The staining intensity was normalized to the background on the same slide and GAG quantification was performed using a custom written Matlab code. Slides from Tr and Db mice were normalized to control mice and only sections of the same staining procedure were compared.

### Statistical analyses

For statistical analyses unpaired t-tests with Bonferoni correction were used (GraphPad Prism5). Error Bars were displayed as ±SD. For all statistical analyzes a *p*-value<0.025 was considered significant.

## Results

### General observations

Average blood glucose levels of ∼250 mg/dl were maintained in all Db mice. Diabetes had no effect on the body weight of the mice; body weights were comparable between groups with mean±SEM for all animals of 22.3±0.92.

### Diabetes induces vertebra bone loss that is mitigated with drug treatment

**Figure 2:**
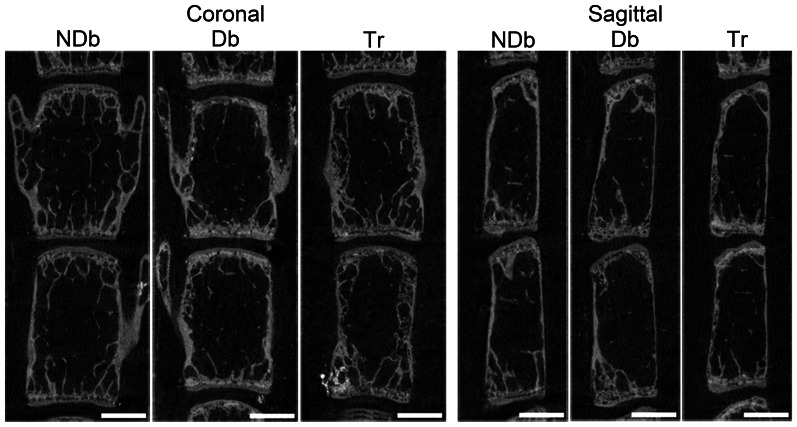
Diabetic induces vertebra bone loss that is mitigated with drug treatment: Representative μCT images of L4–L5 lumbar vertebrae of mid-coronal (left) and mid-sagittal (right) sections.

Db animals had decreased trabecular (Tb) bone volume fraction (BVF, *p* = 0.041) compared to NDb in lumbar vertebrae (Figure 2, Table 1). The decreased BVF was associated with trends of reduced Tb thickness (TbTh, *p* = 0.062) Tb number (TbN, *p* = 0.12) and increased Tb spacing (TbSp, *p* = 0.085). Drug treatment reduced the effect of Db on trabecular bone changes, but BVF was not significantly different to NDb (*p* = 0.17) or Db (*p* = 0.42). The drug treatment prevented the loss of TbTh trending towards greater values (*p* = 0.081) than Db, although no improvements in TbN (*p* = 0.89) or TbSp (*p* = 0.58) compared to Db were noted (Table 1). Analyses of the endplates revealed no differences between groups (data not shown). Similarly, no differences were found within the cortical bone (data not shown). There were also no effects of Db on vertebral length relative to NDb (L4: *p* = 0.155; L5: *p* = 0.992) or Tr (L4 *p* = 0.390; L5 *p* = 0.844).

**Table 1:** Quantitative analyses of vertebral bone.

Condition	NDb	Db	Tr
**Vertebrae length**	3.239±0.323	3.316±0.109	3.305±0.053
**DHI**	0.097±0.01	*0.081±0.010	0.089±0.012
**DWI**	1.893±0.277	2.039±0.349	*1.638±0.239
**TMD (1/mm^3^)**	1088±53	1081±108	1091±61
**BVF (%)**	0.163±0.010	*0.137±0.025	0.149±0.017
**Tb.Th (mm)**	0.039±0.004	“0.036±0.003	0.041±0.003
**Tb.N (1/mm)**	3.959±0.215	3.627±0.665	3.668±0.450
**Tb.Sp (mm)**	0.214±0.015	0.249±0.056	0.235±0.032

Vertebrae length, disc height index (DHI, sagittal), disc wedged index (DWI, sagittal), amount of trabecular (Tb.) bone: Tissue Mineral Density (TMD) Tb. number (Tb.N.) Tb. Spaces (Tb.Sp.) and Bone volume fraction (BVF); Statistics by student t test.

* *p*<0.05;

“ *p* = 0.062; n = 6–8 mice per group.

### Diabetes affects IVD morphology

Disc height in Db mice (2.69 mm ±0.37) was found to be decreased compared to NDb mice (3.18 mm±0.29; *p* = 0.031) while the disc height was maintained in drug treated mice (2.93 mm±0.36; *p* = 0.201). There was a significant reduction in the DHI of Db mice (0.08±0.01) relative to NDb (0.097±0.010; *p* = 0.018), as measured by disc height normalized to vertebrae length (Table 1). However, there was no significant differences between NDb (0.097±0.010) and Tr mice (0.089±0.012; *p* = 0.202). The DWI was found to be lowest in Tr mice (1.64 mm±0.24) and highest in Db mice (2.04 mm±0.35; *p* = 0.032) Yet, no significant changes in DWI between NDb (1.89 mm±0.28) and Db mice (2.04 mm±0.35; *p* = 0.441) or NDb and Tr mice (*p* = 0.1) were observed (Table 1).

**Figure 3:**
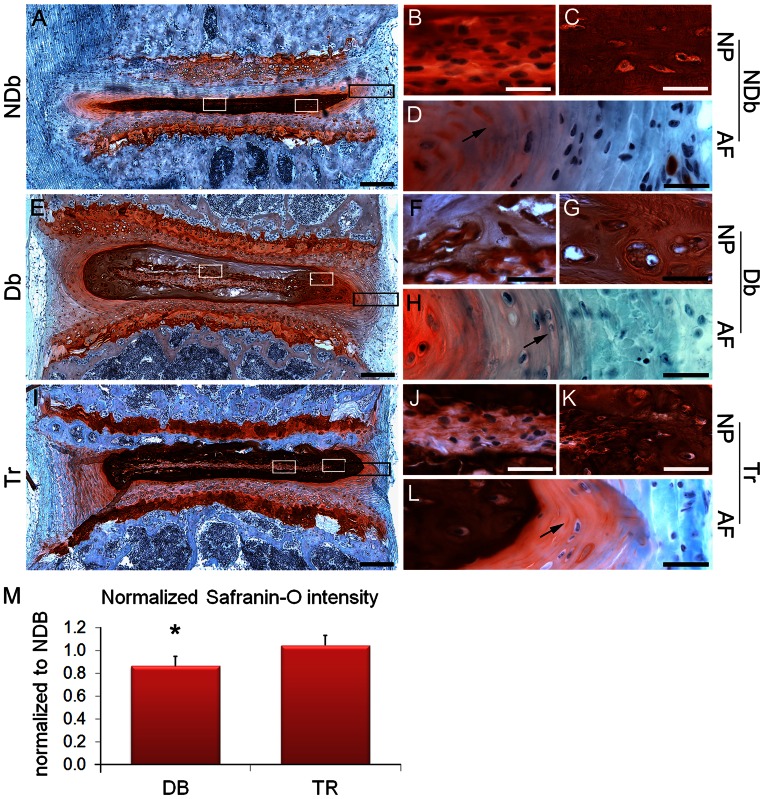
Drug treatment attenuates GAG loss and preserves cell morphology within NP and AF. A–L: representative images of Safranin O staining of NDb, Db and Tr mice. Left: IVD overview images (A,E,I). White boxes (NP) mark magnified areas in the NP of the notochordal band (B,F,J) and the mature GAG rich matrix (C,G,K) containing chondrocytic cells (C+K) in NDb and Tr IVDs or cell clusters in the Db NP (G). Black boxes mark magnified areas of the AF (D,H,L). In NDb and Tr AF cells were mostly fibroblastic and elongated with some small chondrocytic cells (arrows) and distinct, well-formed AF layers (D+L); while the AF of Db IVDs (L) contained many chondrocyte-like cells (arrow). Scale-bars: left = 200 µm; right = 20 µm. M: quantitative analyses of GAG intensity relative to NDb discs; Statistics by student t test * = *p*<0.05; n = 5–6 mice per group.

The morphologically IVDs of NDb and Tr mice appeared thinner than IVDs of Db mice using morphologic techniques (Figure 3A,E,I). In particular the NP of NDb mice was flattened with large notochordal-like cells within a defined ‘notochordal band’ (Figure 3B). The notochordal band was surrounded by a homogeneous mature NP tissue containing small chondrocytic cells and a glycosaminoglycan (GAG) rich matrix (Figure 3C). In the inner and outer AF, cells were mostly fibroblastic and elongated with some small chondrocytic cells and distinct, well-formed AF layers (Figure 3D).

**Figure 4:**
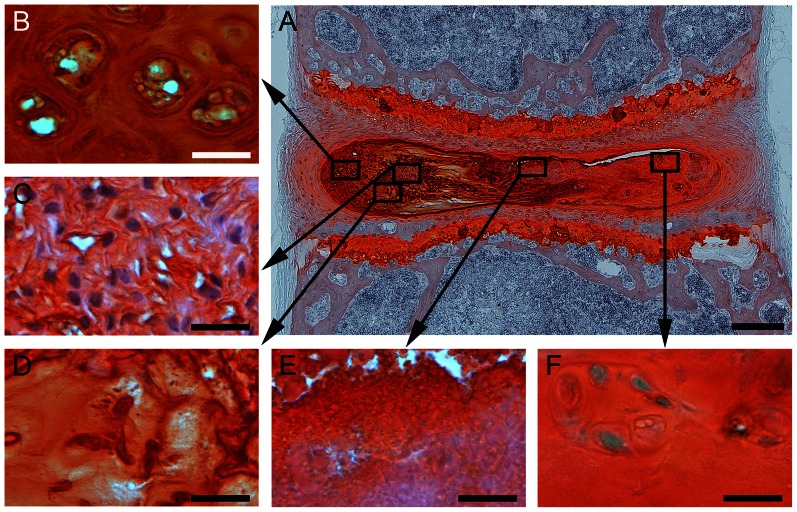
Diabetes leads to changes in NP cell morphology. Representative images of different cell types in the NP of diabetic mice stained with Safranin-O/fast-green. A: overview image, boxes mark region of interest (B–F). B+F: clusters of cells with multiple nuclei and clear cytoplasm; C: remains of the disorganized notochordal band; D: loosened areas of disorganized tissue and cells within the mature matrix; E: areas of unstained deposits of ‘granulation tissue’ with fissures; Scale-bars: A = 200 µm; B–F = 20 µm.

In contrast, Db mice contained marked structural disruption with changes most prominent in the general morphology of the NP. The NP of Db mice appeared thicker with looser mature NP tissue, yet the GAG content was significantly decreased (Db-Tr: *p* = 0.021; norm. to NDb), (Figures 3E–H+4). The notochordal band of the NP was disorganized, thickened, (Figures 3E+4A), and in some cases diminished to small cell clusters (Figure 3F+4C). The margins of the notochordal band were also disorganized and surrounded by a mature NP tissue that was a complex, composite of low cellularity (Figure 3F+4D) with several defining features of dysfunction. Cells were clustered together and appeared to contain multiple nuclei and clear cytoplasm (Figure 3G+4B+4F). The mature NP matrix exhibited less GAG and contained loosened areas of disorganized tissue and cells (Figure 4D). There were also notable areas of unstained deposits of ‘granulation tissue’ with low cell density and containing fissures (Figure 4E). The inner AF of Db mice was mostly populated with chondrocyte-like cells (Figure 3H).

The morphology of Tr mice resembled that of NDb mice with few structural defects restricted to the NP. The NP of the Tr group contained an intact notochordal band although its margins were less clear and shape less flattened. The mature NP tissue also remained largely homogenous with high GAG content and small chondrocyte-like cells, although some clefts and other structural defects were observed (Figure 3I+J+K). The AF region of Tr mice was also similar to NDb with mostly fibroblastic elongated cells and some small chondrocytic cells.

The specific pathological alterations in NP morphology of Db compared to NDb motivated the development of a histomorphometric grading scheme describing early degenerative changes to the mouse NP region (Table 2). More broadly, the NDb mice showed no signs of NP degeneration (score = 0.56±0.1), Db mice showed substantial NP degeneration (score = 6.00±0), and Tr mice showed moderate degeneration (score = 2.67±1.76). However, since there were few alterations to the AF regions only mild loss of IVD height, we contextualize that even the Db animals are only exhibiting early signs of IVD degeneration at this time point.

**Table 2:** Histomorphometric grading scheme for early degenerative changes to the NP of the mouse IVD.

*Status of notochordal band*	
**0**	Distinct and thin
**1**	thicker, disorganized, loss of distinct margins with mature NP matrix
**2**	diminished to cluster of cells
**3**	complete loss of notochordal band

*Chondrocytic cell cluster formation*	
**0**	No cell clusters
**1**	few cell clusters
**2**	substantial cell cloning

*Morphology of mature NP matrix*	
**0**	intensely stained and homogeneous
**1**	homogeneous tissue with loss of staining intensity
**2**	‘loosened matrix’, minor presence of fissures and deposits of ‘granulation’ tissue
**3**	extensive fissures and granulation tissue

### Diabetes results in accumulation of AGEs in IVDs

**Figure 5:**
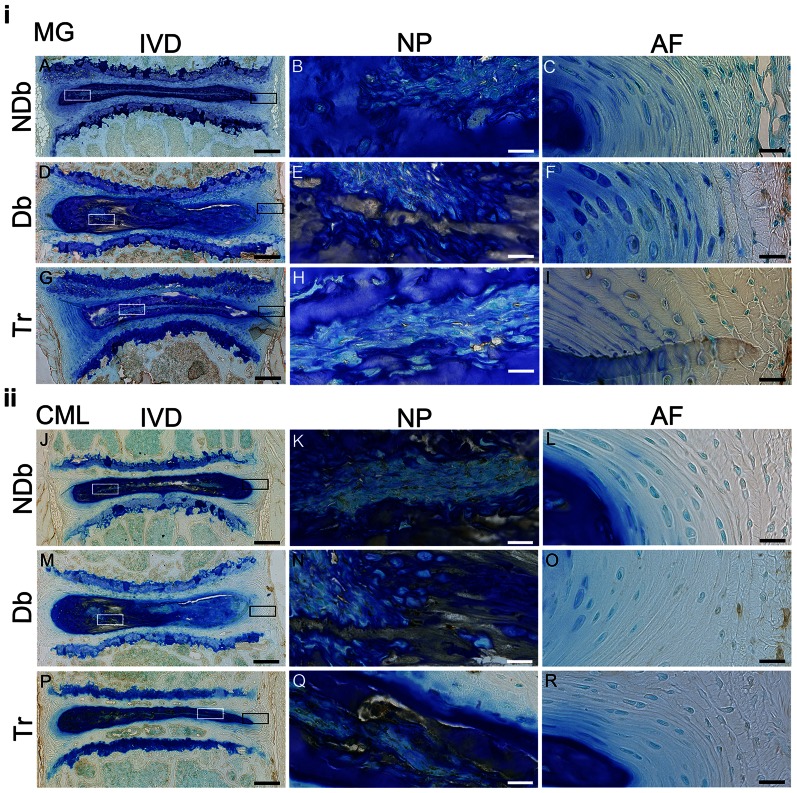
Diabetes causes AGE accumulation in IVD and vertebrae which is partially mitigated after treatment. Representative images of immunohistochemistry for MG (top, A–I) and CML (bottom, J–R) of NDb, Db and Tr IVDs (left: 5× magnification); boxes mark 40× magnified area of NP (white box; MG = B,E,H and CML = K,N,Q) and AF (black box; MG = C,F,I and CML = L,O,R). Scale-bars: left (IVD) = 200 µm; right (NP+AF) = 20 µm.

The amount of stainable AGEs (MG) was increased in diabetic mice. Most of the increase was observed within the NP, where immunopositive deposits accumulated in areas of very low cellularity. There was moderate increase in MG positivity in Tr mice as compared to NDb and weak immunostaining above background was present in NDb mice (Figure 5i). MG accumulation within the extracellular matrix of bone was not different between Db and Tr mice, but MG in Db and Tr mice was greater than in NDb mice. While there was less MG-positive material in the AF, slightly more positive cells and matrix were observed in the outer AF of Db and Tr mice (Figure 5i). The accumulation of CML was increased in vertebrae of both, Db and Tr mice, while NDb mice showed the least CML staining. No apparent differences between NDb, Db and Tr mice with respect to CML accumulation was observed within the NP or AF (Figure 5ii).

### AGE accumulation is associated with increased TNFα and enhanced catabolism

**Figure 6:**
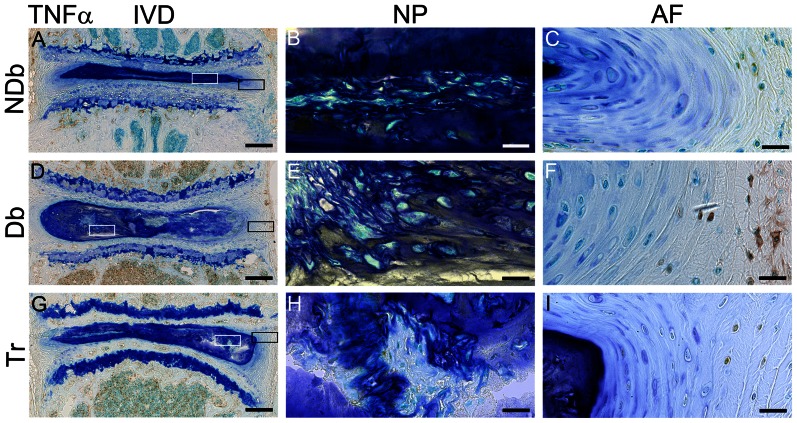
AGE accumulation is associated with increased TNFα activity. Representative images of immunohistochemistry for TNFα of NDb, Db and Tr discs (A,D,G; 5× magnification,). Boxes mark 40× magnified areas of NP (white box; B,E,H); and AF (black box; C,F,I). Scale-bars: left (IVD) = 200 µm; right (NP+AF) = 20 µm.

**Figure 7:**
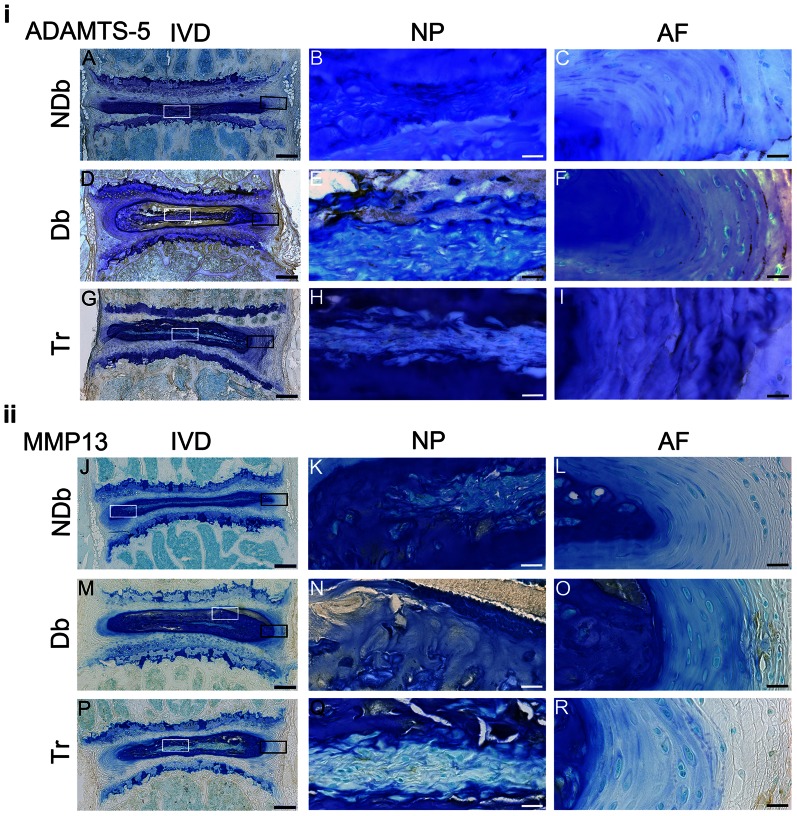
Catabolic response to AGE accumulation in Db mice is attenuated in Tr mice. Representative images of immunohistochemistry for catabolic proteins ADAMTS-5 (top A–I) and MMP13 (bottom, J–R) of NDb, Db and Tr mice (left: 5× magnification). Boxes mark 40× magnified areas of NP (white box; ADAMTS-5 = B,E,H; MMP13 = K,N,Q); and AF (black box; ADAMTS-5 = C,F,I; MMP13 = L,O,R). Scale-bars: left (IVD) = 200 µm; right (NP+AF) = 20 µm.

Immunostaining for the pro-inflammatory cytokine TNFα was observed most strongly in Db mice. Similar to MG accumulation, TNFα was less prominent in Tr compared to Db discs, and NDb animals had the least amount of staining (Figure 6A). TNFα immunostaining was most strong in the vertebrae and AF regions in Db and Tr groups. The NP exhibited positive immunostaining in Db animals, particularly in the deposit tissue. Immunostaining of Tr mice spines showed reduced expression of the catabolic proteins disintegrin-and-Metalloproteinase-with-Thrombospondin-Motif (ADAMTS-5) and matrix metalloproteinase13 (MMP13) as compared to Db mice (Figure 7). ADAMTS-5 was highly expressed throughout Db mice spines, particularly in the extracellular matrix of bone and NP. Tr mice showed only moderate ADAMTS-5 expression and NDb mice only had a minor amount of ADAMTS-5 positive staining (Figure 7i). Expression of MMP13 appeared faint in all groups; yet, increased MMP13 expression was evident in Db discs compared to Tr and NDb discs (Figure 7ii).

## Discussion

This study indicated that diabetes induced in young mice resulted in IVD degeneration and bone loss in vertebrae. This is the first study to carefully characterize these morphological changes in the NP with diabetes and to demonstrate an associated with AGE accumulation and increased catabolism. Investigation of spinal structures demonstrated **1**. this *in-vivo* diabetes model was associated with early structural alterations representative of early IVD degeneration, and **2**. diabetes, a systemic disease, can influence the entire spine organ system. This is also the first study to demonstrate that oral ingestion of medications can be beneficial in treating spinal pathologies. A combination of an anti-inflammatory and a drug that blocks the intracellular formation of methylglyoxal (an AGE) was given orally. These drugs reduced the accumulation of AGEs in treated diabetic mice, suggesting that the accumulation of AGEs was associated with diabetic degenerative changes in the spine. In particular, diabetes was associated with loss of disc height, increased DWI, decreased vertebral bone mass, decreased IVD glycosaminoglycan content and morphological alterations that were most prominent in the NP region. NP structural alterations involved disruption of the notochordal band and defects in the mature NP matrix including clefts and deposition of granulation tissue that stained positive for AGEs, TNFα, MMP-13 and ADAMTS-5. Drug treatment targeted to inhibit oxidative stress, inflammation and AGE-accumulation prevented or reduced many of the pathological effects diabetes had on vertebrae and IVD.

The drug treatments included a combination of the anti-inflammatory drugs PPS and PYR, an inhibitor of MG formation, and EN an angiotensin converting enzyme inhibitor. PPS is an FDA-approved, oral medication (Elmiron®) that is used in the treatment of interstitial cystitis and has an excellent, long-term safety profile in humans. It also prevents renal lesions and preserves kidney function in old diabetic mice and in 5/6 nephrectomized rats, and inhibits the pro-inflammatory actions of TNFα [37], [38]. PPS can promote chondrogenesis in bone marrow derived mesenchymal precursor cells [39] and was demonstrated to show cartilage improvement in osteoarthritis patients in an off-label clinical trial [40]. We propose that PPS may be an effective treatment for IVD degeneration. PYR is a vitamin B6 vitamer which is converted to the biologically active form of vitamin B6 and in lower dosage ingested with the regular diet. The natural AGE blocker acts via 3 mechanisms to inhibit AGE formation. First, by blocking the oxidative degradation during the Maillard reaction. Second, by removing toxic carbonyl products derived from both glucose and lipids such as glyoxal and methylglyoxal (MG). Third, by sequestering reactive oxygen species [33], [41]. A variety of preclinical studies indicate that PYR improves kidney structure and function and has been tested for clinical utility in the treatment of diabetic nephropathy [42]. EN, an angiotensin converting enzyme inhibitor, is the standard treatment of diabetes complicated by kidney disease hypertension. EN reduces the rate of decline in kidney function in patients with diabetic nephropathy and has good long term tolerance [43]. EN (standard of care treatment of diabetic patients with kidney disease) and PYR (natural AGE inhibitor) have been shown to be effective for treating diabetes. PPS has also been shown to reduce TNFα-induced changes and to block the development of diabetic nephropathy in mice. By combining these potent drugs into one cocktail we expected the effect to be additive, since the triple-treatment acts through different pathways, namely by counteracting the de-novo formation of AGEs intracelllularly and reducing AGE-mediated inflammatory changes. Future investigations are necessary to more precisely inform the mechanisms for AGE-induced spinal pathologies in diabetics with renal disease and their interventions.

AGEs are known to modulate several intracellular and extracellular structural and pro-oxidant effects, including inflammation and alterations of proteins and lipids [24]. In long-lived proteins, such as collagen, structural changes from AGE crosslinking accumulate over time, leading to stiffening of collagen rich tissues, including arteries, skin and cartilage [44], [45]. AGE accumulation alters the mechanical behavior of AF tissue, resulting in decreasing Poisson's ratio and increased tissue fragility [27], [28].

Diabetes increased the abundance of AGEs and spinal tissue pathology. Stable hyperglycemia, which was induced by Streptozotocin injections in both Db and Tr groups, led to increased generation of AGEs. In addition to the AGEs generated by hyperglycemia, AGEs present in the standard chow contributed to the high abundance of AGEs in these mice [24]. CML abundance was found in both, Db and Tr discs. CML positive matrix and cells were also found in NDb mice, especially within the NP, as has been noted in other tissues [29]. The CML baseline expression in NDb mice is likely to result from the high content of AGEs found in standard chow that all mice were fed. Further, the slow metabolism within the NP in combination with a half-life of 12±2.0 years for aggrecan [26], [46] may also have led to the accumulation of CML within healthy NPs. Only minor CML expression was observed within vertebrae of NDb mice, possibly due to constant matrix remodeling in cartilaginous and boney tissues during adolescence.

Despite accumulation of AGEs, treatment with anti-AGE and anti-inflammatory drugs inhibited MG and mitigated a number of degenerative processes in the spine. MG is a highly reactive α-dicarbonyl that reacts with lipids and proteins causing irreversible alterations [47]. MG is among the best-known glycation agents linked to diabetic or age-related cell injury, and is known to promote weight gain, adiposity, and metabolic changes in mice [22]. The current results revealed decreased accumulation of MG immuno-staining in Tr mice, and are suggestive that controlling creation and effects of MG may be more important than controlling CML in managing diabetes-related spinal pathologies. Many authors have shown that the receptor for AGEs (RAGE) is induced by AGEs and RAGE levels are high in both animals and humans which are diabetic (24). RAGE expression is expected to be closely associated with the levels of AGEs, and there are also several receptors for AGEs (24). Important future studies exploring mechanisms of AGE accumulation and specifically targeted interventions are a large area that we believe are strongly motivated by the current work. However, the current study focuses on the impact of diabetes on AGE accumulation and the corresponding inflammatory and catabolic responses of the IVD. We therefore measured TNF-α because of its well-known involvement in IVD disease, and the fact that PPS blocks TNFα-mediated changes [38].

The present *in vivo* study supports the concept that diabetes is associated with increased AGE accumulation and up-regulation of the inflammatory cytokine TNFα. The presence TNFα presumably accelerates aggrecan degradation and turnover by inducing up-regulation of the catabolic proteins ADAMTS-5. The up-regulation of ADAMTS-5 could lead to the significant loss of GAG observed in IVDs of Db mice in the current study since ADAMTS-5 is known to be a major aggrecanase in mouse cartilage [48], and aggrecan is the major GAG within the IVD. In addition, an *in vitro* study performed on hyperglycemic bovine NP cells revealed that the direct administration of AGEs to NP cells causes a down-regulation of aggrecan [49].

The structural changes in diabetic mice IVDs were notable and particularly prominent in the NP. Plastic embedded mouse specimens provided high quality resolution that allowed thorough characterization of early structural changes in the experimental IVDs. The IVDs of healthy NDb animals contained a NP with a centrally located and flattened notochordal band that was surrounded by mature NP matrix lower in cell density and rich in GAG. Several of these healthy NP morphological features in the mice were published previously [50], although the morphology of the notochordal band is a feature that can be difficult to describe and characterize in mice without plastic embedding, refined histological methods and high magnification.

The NP of Db mice were severely compromised and several of these morphometric characteristics are newly described. Specifically, the notochordal band was disrupted, thickened, or broken into smaller cell clusters, and the margins between the notochordal band with mature NP matrix were less apparent. The mature NP matrix area exhibited a loss of GAG and notable structural defects that included clefts. Another new observation is the accumulation of tissue deposits in the NP that stained positively for MG, TNFα, MMP-13 and ADAMTS-5. These findings complement studies performed on aging sand-rats which demonstrated that diabetes is associated with increased IVD cell death and that diabetes can lead to alterations in bone mineral density and increased endplate calcification [11], [13], [18].

IVDs of Tr mice exhibited morphology and immunostaining characteristics that were closer to those found in NDb than Db mice, and also maintained disc height. The localized structural defects observed in the NP of NDb, Db and Tr animals are suggestive of 2 important hypotheses that require additional testing. First, can the IVD degeneration induced by diabetes initiate the localized deposits of granulation tissue in the NP that are high in AGEs and induce a pro-inflammatory cascade? Second, can the oral medications that inhibit accumulation of AGEs, reactive oxygen species and pro-inflammatory cytokines inhibit certain degenerative changes to the IVD? The apparent differences in IVD dimensions between quantitative μCT measurements and qualitative observations made when reviewing the histological sections (i.e., diabetic specimens appear to have greater IVD height) are related to planes of observation and differences altered spinal curvature for the Db group. Histological specimens were processed with coronal sections, while disc dimensions based on μCT were calculated according to established protocols [34], [35] using mid-sagittal sections which also documented alterations in DWI.

The reduction of MG immunostaining suggests that the improvements seen in the spine resulted from the oral drugs is directly associated with their action on matrix and cells in the spine. However, it is possible that the observed benefits of treatment are associated with systemic improvements in the OS and inflammation.

To our knowledge, this is the first study to show that DM induced pathological changes in lumbar IVDs and vertebrae and that an important mechanism for these changes is AGE accumulation and increased inflammation. The specific localized accumulation of morphological defects and deposition of pro-inflammatory tissues in the NP may be important factors in the initiation of IVD degeneration more broadly than in this mouse DM model. This conclusion is strengthened by the fact that loss in disc height, decreased vertebral bone mass, altered NP morphology and reduced GAG content was prevented by treatment with drugs that reduce inflammation and AGEs. From these observations we conclude that the oral ingestion of AGE inhibitory and anti-inflammatory drugs provide a safe and efficacious treatment that may slow the progression of diabetes-induced changes within the spine, and this is also the first study to demonstrate effective treatment of spine diseases via oral medications in young animals. These findings have potentially broad impact due to the tremendous prevalence of both diabetes and back pain, as well as the known accumulation of AGEs in aging IVDs.
