# Correction: Combined Anti-Inflammatory and Anti-AGE Drug Treatments Have a Protective Effect on Intervertebral Discs in Mice with Diabetes

**DOI:** 10.1371/journal.pone.0119112

**Published:** 2015-03-05

**Authors:** The PLOS ONE Staff

There are errors in the Funding section. The correct funding information is as follows: Research reported in this publication was supported by grants from the National Institute of Arthritis and Musculoskeletal and Skin Diseases of the National Institutes of Health (R01 AR057397 and R01 AR064157), the National Institute of Health/National Institute of Diabetes and Digestive and Kidney Diseases (NIH/NIDDK; RO1 AG023188), the Juvenile Diabetes Research Foundation (JDRF; 17-2008-1041), and the North American Spine Society (basic research).

## References

[1] Illien-Junger S, Grosjean F, Laudier DM, Vlassara H, Striker GE, Iatridis JC (2013) Combined Anti-Inflammatory and Anti-AGE Drug Treatments Have a Protective Effect on Intervertebral Discs in Mice with Diabetes. PLoS ONE 8(5): e64302 doi:10.1371/journal.pone.0064302 23691192 10.1371/journal.pone.0064302 PMC3656842

